# Dauriporphine inhibited lung cancer cell viability, motility, and energy metabolism through the miR-424-5p/MAPK14 axis

**DOI:** 10.1186/s41065-025-00473-w

**Published:** 2025-06-11

**Authors:** Yan-Jia Du, Jin-Peng Lv, Yao Fu, Meng Lan, Jing-Feng Li, Hui Zhang, Nan Wu

**Affiliations:** 1 Department of Chinese Medicine and Health Care, Changchun Humanities and Sciences College, No. 1488, Boshuo Road, Jingyue National Hi-Tech Industrial Development Zone, Changchun, 130117 Jilin Province China; 2 Jilin Northeast Asia Biotechnology Development Co., Ltd, Room 16-3-105, Luwafangfu’ao I, Jingyue Development Zone, Changchun City, Jilin Province 130033 China; 3 Institute of Special Animal and Plant Sciences, Chinese Academy of Agricultural Sciences, Changchun, 130022 China; 4 College of Pharmacy, Changchun University of Chinese Medicine, Changchun, 130117 China

**Keywords:** Anti-lung cancer treatment, Dauriporphine, Cell viability, Cell motility, Energy metabolism

## Abstract

**Background:**

Dauriporphine is a major ingredient of *Manispernum daericum* DC., which has been demonstrated to show wide anti-tumor activities. miR-424-5p, as a regulator of lung cancer, was hypothesized to serve as the therapeutic target for dauriporphine This study evaluated the potential of dauriporphine in treating lung adenocarcinoma and revealed the underlying molecular mechanism.

**Results:**

The anti-tumor effect of dauriporphine on lung adenocarcinoma was assessed in A549 cells, and it was found that dauriporphine significantly inhibited the viability of A549 cells in a concentration-dependent manner with the half maximal inhibitory concentration (IC_50_) value of 10.57 µM. Dauriporphine induced decreasing cell growth, motility, and energy metabolism, indicating the anti-tumor effect of dauriporphine on A549 cells. Dauriporphine inducing elevated miR-424-5p levels, while silencing miR-424-5p significantly recovered cell viability, migration, and energy metabolism of A549 cells. Mitogen-activated protein Kinase 14 (MAPK14) was negatively regulated by miR-424-5p, and the knockdown of MAPK14 could reverse the protective effect of miR-424-5p on dauriporphine-treated A549 cells.

**Conclusion:**

Dauriporphine inhibited cell growth, metastasis, and glycolysis-related energy metabolism of lung adenocarcinoma cells via modulating miR-424-5p/MAPK14 axis. Dauriporphine can be considered in drug development for lung adenocarcinoma.

**Clinical trial number:**

Not applicable.

**Supplementary Information:**

The online version contains supplementary material available at 10.1186/s41065-025-00473-w.

## Introduction

Lung cancer ranks in the top position in the causes of cancer-related deaths [1]. The onset of lung cancer involves complex factors, including environmental factors, smoking, and occupational factors. Among various subtypes of lung cancer, non-small cell lung cancer showed a high percentage of new cases, where lung adenocarcinoma is considered the most common subtype [2, 3]. Clinically, surgery, chemotherapy, and radiotherapy are the main means for the treatment of lung adenocarcinoma [2, 4]. However, there are several disadvantages disclosed, such as resistance, cytotoxicity, and other adverse reactions. With the development of traditional Chinese medicine (TCM), there have been several herbal prescriptions developed for lung adenocarcinoma [5–7]. From the perspective of Chinese medicine, the onset of lung adenocarcinoma is associated with the deficiency of positive qi and invasion of toxicity, which results in poor lung circulation and internal retention of body fluids [8, 9]. Over time, phlegm, blood stasis, and the generation of toxins would occur. Due to the characteristics of multiple targets and complex components, TCM showed significant advantages of lower resistance [10]. Network pharmacology always identifies several therapeutic targets of one TCM herb or active ingredient. The complex regulatory network could avoid the drug resistance compensation mechanism caused by excessive activation of a single target.

Alkaloid is one of the major active ingredients in *Manispernum daericum* DC. and is also the earliest species isolated and identified [11, 12]. Alkaloids isolated from *Manispernum daericum* DC. mainly characterized as isoquinoline alkaloids, including Menispermine, Phenolic alkaloids, and other isoquinoline derivatives with diverse structures and complex structures with multiple rings and substituents [11]. The extraction of *Manispernum daericum* DC. has been demonstrated to show significant pharmacological activities of antitumor, anti-inflammation, liver protection, hypoglycemic, and cardioprotective effects [13, 14]. Considering the difference in the chemical structure of alkaloids, different kinds of alkaloids isolated from *Manispernum daericum* DC. might display distinct effects. For example, dauricine has been demonstrated to regulate the progression of vascular endothelial inflammation, prostate cancer, and pancreatic cancer [15–18]. The antitumor effects of daurisolin have also been revealed in glioma, esophageal squamous cell carcinoma, and bladder cancer [19–21]. The pharmacological activities of these two isolations have been revealed associated with related therapeutic targets. For instance, the protective effect of dauricine on osteoclast genesis was demonstrated to display through the NF-kappaB and NFATc1 pathway, which also mediated its anti-inflammatory activity [22–24]. Dauriporphine has been identified as a major ingredient for the antitumor activity of *Polygonatum sibiricum* [25]., but specific validation and deep mechanisms have not been investigated.

Recent studies have noticed the function of microRNAs (miRNAs) in human cancers. miRNAs have also been reported to participate in the pharmacological activities of TCM herbs. For example, the antitumor effect of dauricine on hepatocellular carcinoma cells was revealed to involve miR-199a [26]. miR-424-5p was identified as a cancer-related miRNA and has also been revealed to play a critical role in the progression of lung cancer. miR-424-5p was demonstrated to regulate therapy resistance and metastasis [27, 28]. Additionally, the sponging of miR-424-5p with lncRNA LINC00641 served as a tumor suppressor of non-small cell lung cancer [29]. A network pharmacology study identified MAPK14 as a therapeutic target of dauriporphine [25], and MAPK1 was also demonstrated to show a close association with the onset and development of lung cancer [30–32]. The target relationship between miR-424-5p and MAPK14 was predicted from public databases and has been confirmed in lung injury associated with the risk of lung cancer [33]. Therefore, the miR-424-5p/MAPK14 axis was hypothesized to mediate the potential anti-lung cancer effect of dauriporphine, which lacks direct evidence.

This study focused on the potential anti-tumor effect of dauriporphine on lung cancer with the help of in vitro cell experiments, aiming to uncover the underlying mechanism and to provide more references for developing novel therapeutic strategies for lung cancer.

## Materials and methods

### Cell culture and cell treatments

A549 cells purchased from ATCC were maintained with DMEM culture medium (Invitrogen, USA) supplemented with 10% FBS and 1% penicillin and streptomycin. Cells were incubated at 37 °C with 5% CO_2_ until reaching the logarithmic growth stage. Cells were treated with 0–20 µM dauriporphine (purity > 98%, MedChemExpress, USA).

### Cell transfection

Cells were transfected with miR-424-5p inhibitor and corresponding negative control to silence miR-424-5p and transfected with small-interference RNA of MAPK14 to knock down MAPK14. Cells were seeded into 6-well plates at a density of 3 × 10^6^ cells/well supplied with 125 µL FBS-free culture medium. Cell transfection was conducted at room temperature using Lipofectamine 2000 (Invitrogen, USA). The transfections were utilized with a ratio of 1:1 to Lipofectamine 2000. Transfection efficiency was assessed after 48 h of transfection through the expression of miR-424-5p and MAPK14 using qPCR.

### Real-time quantitative PCR

Total RNA was extracted from cells using Trizol reagent (Invitrogen, USA) and evaluated for concentration and purity using NanoDrop 2000 Spectrophotometer (NanoDrop Technologies, USA). The isolated RNA was reverse transcribed into cDNA using the miRcute miRNA cDNA kit (Tiangen, China) and the High-Capacity cDNA Reverse Transcription Kit (Applied Biosystems, USA).

PCR amplification was performed on the 7500 PCR system (Applied Biosystems, USA) with the help of SYBR Green kit (Tiangen, China). The sequence of experimental primers has been summarized in Table S1. The 2^−ΔΔCT^ method was performed to calculate the relative expression levels of miRNA and mRNAs. U6 and GAPDH were employed as internal references for miRNA and mRNA, respectively.

### Cell viability assay

Cells after corresponding transfection (1 × 10^3^ cells/well) were seeded into 96-well plates supplied with 10% FBS-containing DMEM culture medium. To evaluate the effect of dauriporphine, 10 µM dauriporphine was added to the incubating wells. The plates were incubated at 37 °C for 24, 48, and 72 h, followed by adding a CCK8 reagent (Multiscience, China). The absorbance at 450 nm at each time point was detected with a microplate reader, and the OD_450_-time curve was employed to evaluate cell viability.

### Cell motility assay

Cells after corresponding transfection (5 × 10^4^ cells/well) were seeded into the upper chamber of 24-well Transwell plates supplied with an FBS-free culture medium and 10 µM dauriporphine. The bottom chambers were filled with a completed culture medium containing 10% FBS. The plates were maintained at 37 °C for 48 h to allow cells mitigated to the bottom chamber. Cells in the upper chamber were removed after incubation, and the subsurface of the upper chamber was fixed (by paraformaldehyde) and stained with 0.1% crystal violet. Cell counting was conducted with an optical microscope (Olympic MX-50, Japan), and five fields of each treatment were randomly captured. For the invasion assessment, the upper chamber was pre-coated with Matrigel.

### Energy metabolism evaluation based on ATP, LDH, glucose consumption, and lactate production

Cells (1 × 10^5^ cells/well) were seeded into 96-well plates and treated with 10 µM dauriporphine for 24 h. The culture medium was collected after cell incubation for the following analysis.

The collection incubation solution was mixed with Glucose Assay Reagent (Beyotime, China) and vortexed to even. The mixture was centrifugated at 5000 g for 30 s, and the absorbance at 630 nm was measured to evaluate the concentration of glucose. The consumption of glucose was calculated by the difference between the cell culture and the blank culture medium.

The ATP levels were detected with the employment of ATP Bioluminescence Assay Kit CLS III (Roche Applied Science, Germany). Briefly, Cells were lysed with Assay Buffer and centrifugated at 10,000 g for 5 min. The supernatant was further mixed with the Assay Buffer and incubated with the detection kit in the dark at room temperature for 30 min. The absorbance at 570 nm was detected by a microplate reader to evaluate the ATP levels.

The activity of LDH was assessed with the Lactate dehydrogenase detection kit (Beyotime, China). Cells were incubated with an FBS-free culture medium and LDH release regent for 1 h at 37 °C. Then, the mixture was centrifugated at 500 g for 5 min. The supernatant was mixed with the detection kit and incubated in the dark for 30 min. The absorbance at 490 nm was detected by a microplate reader to evaluate the activity of LDH.

Cells were incubated with an FBS-free culture medium for 1 h, the supernatant was collected for the analysis of lactate production. The collected supernatant was mixed with the Lactate Colorimetric Assay Kit II (Biovision, USA) and incubated in the dark for 30 min. The absorbance at 450 nm was detected with a microplate reader.

The LDH, ATP, glucose consumption, and lactate production levels were presented as percentages of control by normalization.

### Luciferase reporter assay

The binding sites between miR-424-5p and MAPK14 were predicted from the Starbase database. The wild-type and mutant-type vectors were established by cloning wild-type or mutated sequences into the pmiR-RB-REPORT (Ribobio, USA). Cells (2 × 10^4^ cells/well) were co-transfected with established vectors and miR-424-5p mimic, inhibitor, or negative controls in the 24-well plates for 48 h. The luciferase activity of MAPK14 was analyzed by the dual-luciferase reporter assay system (Promega, USA) with Renilla as the internal reference.

### Statistical analysis

All experiments were performed in triplicate independently with three repeated measurements of each. Data were expressed as mean ± SD., and a difference comparison was conducted with the student’s t-test and one-way ANOVA using GraphPad Prism 9.0. Statistical significance was indicated by *P* < 0.05.

## Results

### Dose-inhibition of A549 cell viability by dauriporphine

**Fig. 1:**
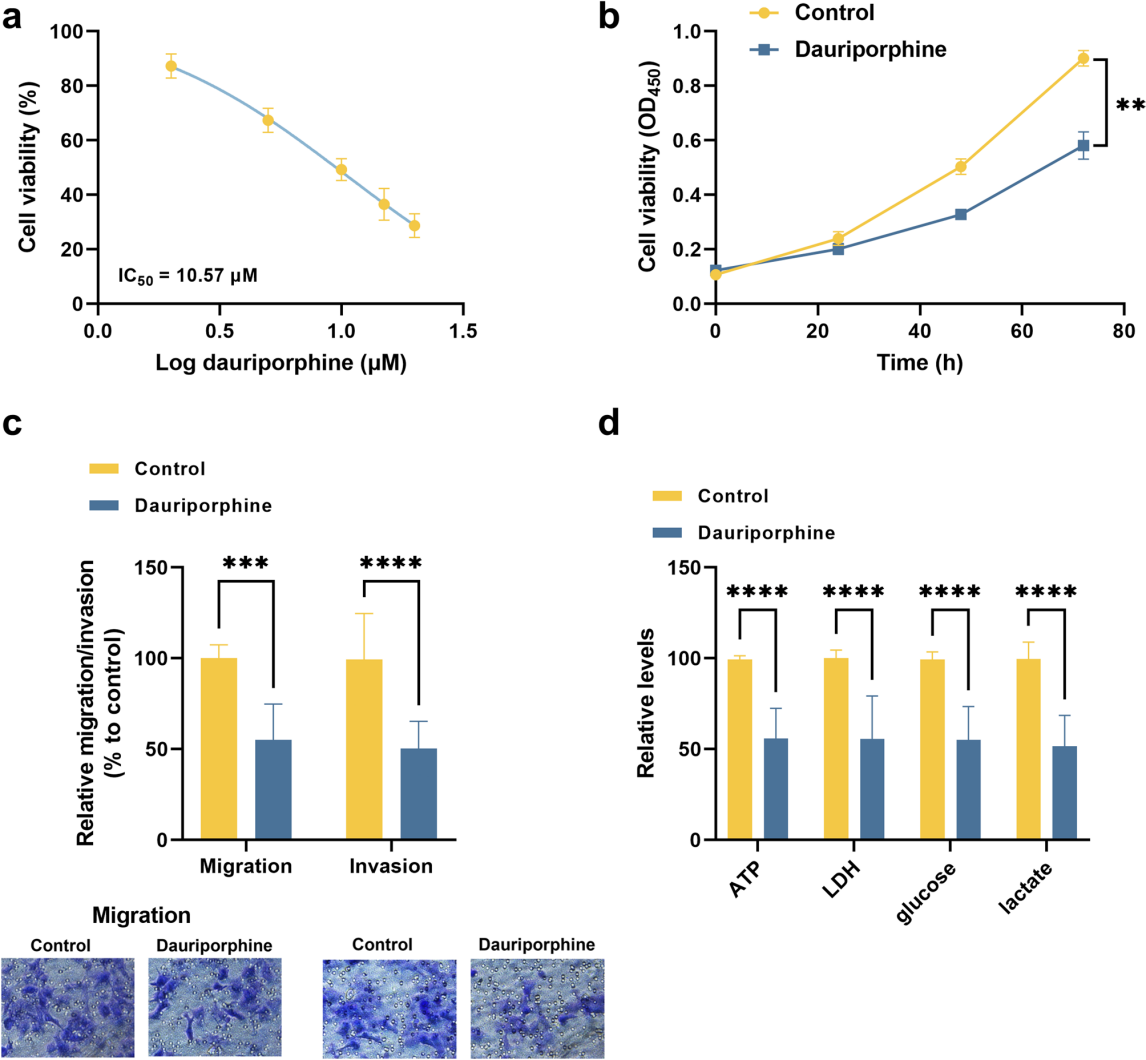
Anti-tumor effect of dauriporphine on A549 cells. (**a**). concentration-dependent inhibition of A549 cells by dauriporphine. IC_50_ was calculated by non-linear regression analysis based on the logarithm of dauriporphine concentration.b-d. Inhibition of cell viability (**b**), motility (**c**), and energy metabolism (**d**) by dauriporphine. ^**^P < 0.01, ^***^P < 0.001, ^****^P < 0.0001 compared by student’s t-test (*n* = 3). Control: without any treatments on the A549 cell; dauriporphine: A549 cells treated with 10 µM dauriporphine

In the presence of 0, 5, 10, 15, and 20 µM dauriporphine, the viability of A549 cells decreased with the increasing concentration, and an IC_50_ value of 10.57 µM was obtained (Fig. 1a). A concentration close to IC_50_, 10 µM was selected for the following experiments. It was found that dauriporphine showed significant inhibitory effects on viability (Fig. 1b), motility (Fig. 1c), and energy metabolism (Fig. 1d), suggesting the anti-tumor effect on A549 cells, and the underlying mechanism was further investigated.

### Silencing miR-424-5p alleviated the inhibitory effect of dauriporphine on A549 cells

miR-424-5p was significantly downregulated in lung cancer cells (A549 and H1299 cells) relative to normal HBE cells (Figure S1a). Overexpressing miR-424-5p (Figure S1b) significantly suppressed the viability (Figure S1c), migration (Figure S1d), and invasion (Figure S1e), indicating its tumor suppressor role.

**Fig. 2:**
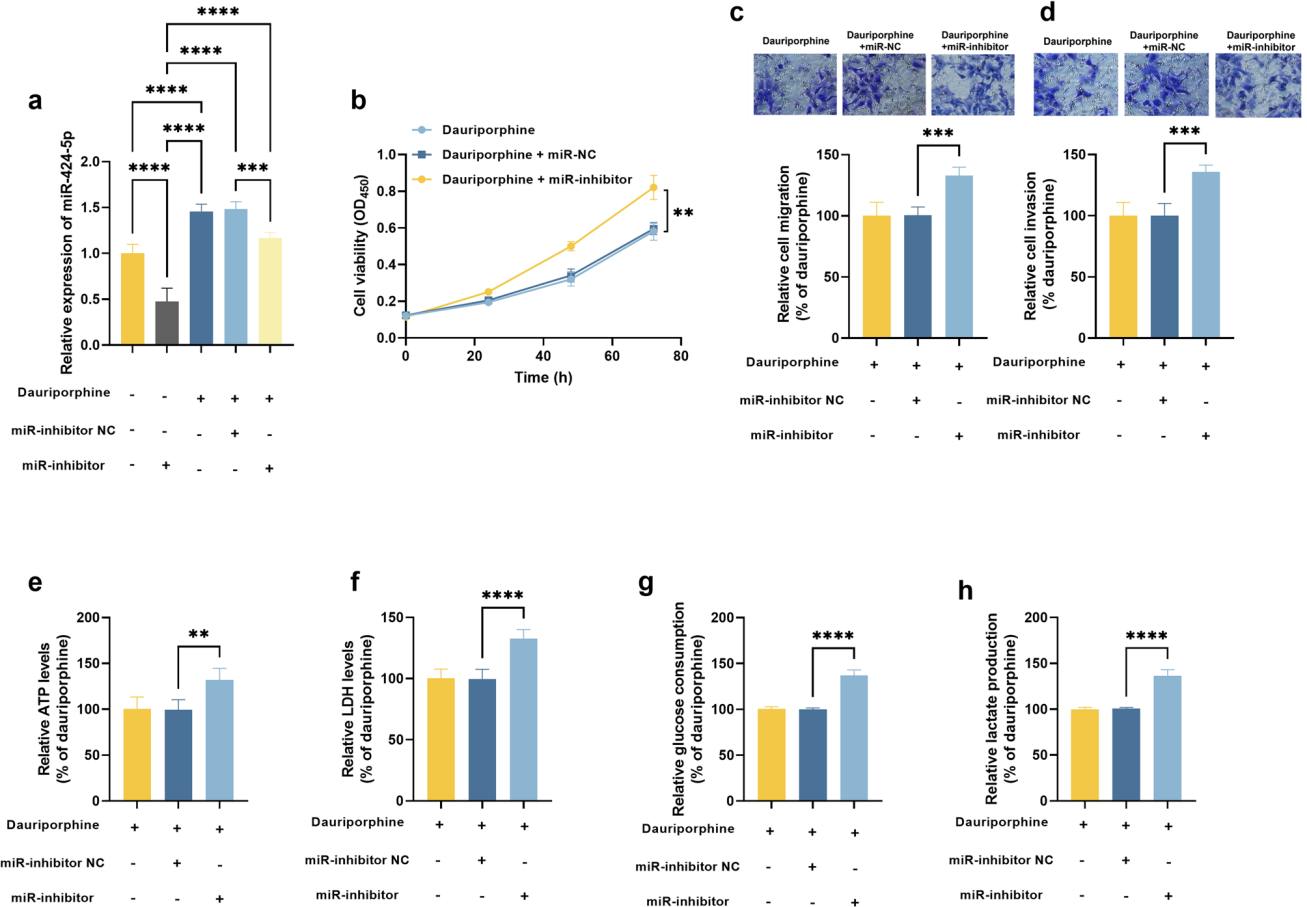
Involvement of miR-424-5p in the anti-tumor effect of dauriporphine. Expression of miR-424-5p (**a**). b-f. Cell viability (**b**), migration (**c**), invasion (**d**), ATP level (**e**), LDH level (**f**), glucose consumption (**g**), and lactate production (**h**) regulated by miR-424-5p in dauriporphine-treated A549 cells. ^**^P < 0.01, ^***^P < 0.001, ^****^P < 0.0001 compared by one-way ANOVA (*n* = 3). dauriporphine: 10 µM dauriporphine; miR-inhibitor NC: 20 nmol miR-424-5p inhibitor negative control; miR-inhibitor: 50 nmol miR-424-5p inhibitor

Additionally, significant upregulation of miR-424-5p was observed in dauriporphine-treated A549 cells, which was suppressed by its inhibitors (Fig. 2a). Silencing miR-424-5p recovered the viability of dauriporphine-treated A549 cells (Fig. 2b), promoted cell migration (Fig. 2c) and invasion (Fig. 2d), and enhanced cell energy metabolism, indicated by the increasing levels of ATP (Fig. 2e) LDH (Fig. 2f), glucose consumption (Fig. 2g), and lactate production (Fig. 2h).

### miR-424-5p mediated the anti-tumor effect of dauriporphine on A549 cells through targeting MAPK14

**Fig. 3:**
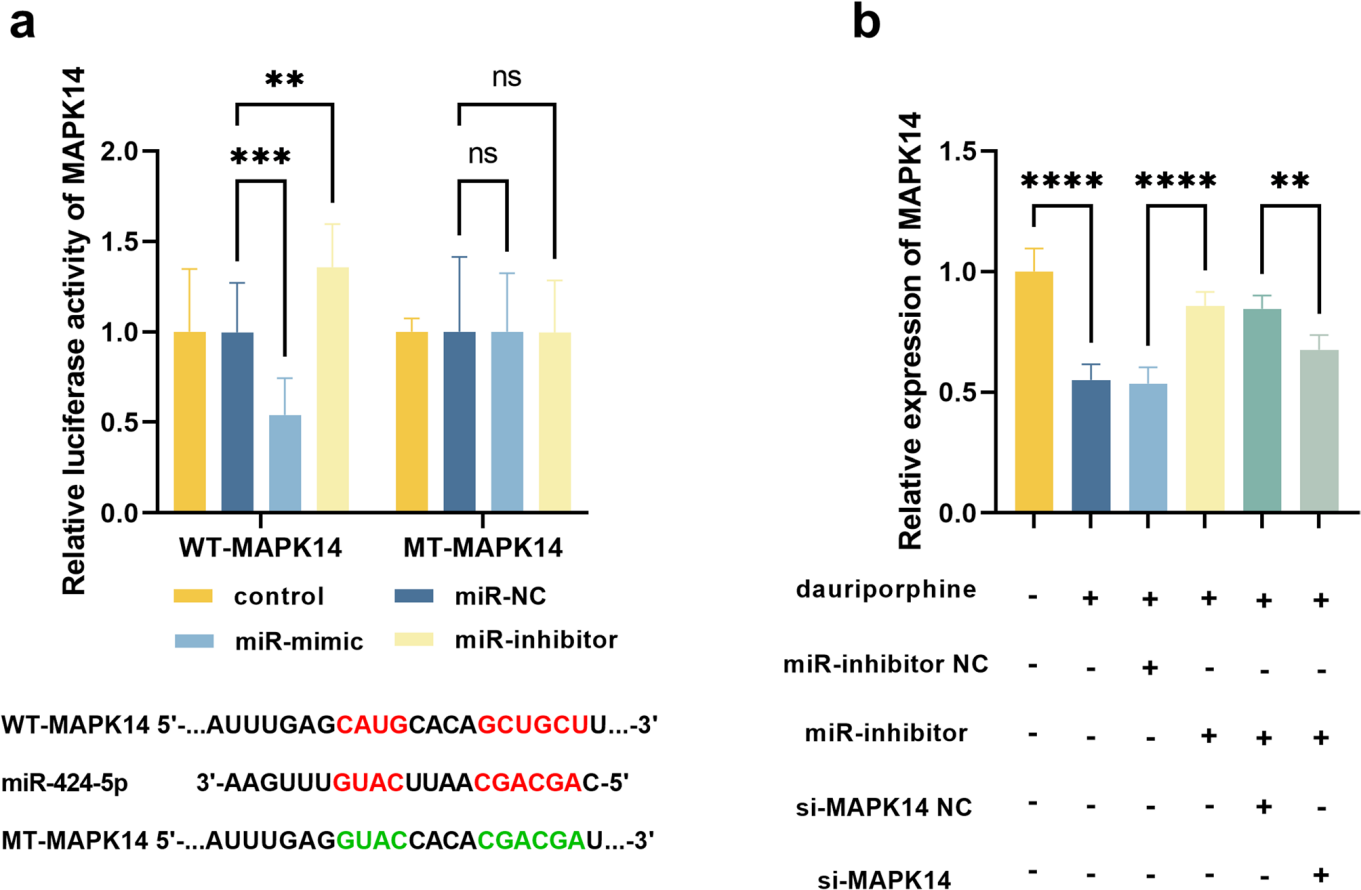
Targeting relationship between miR-424-5p and MAPK14. Binding sites and luciferase activity of MAPK14 regulated by miR-424-5p (**a**). The red base pairs were matched, and the green base pairs were mutants. Expression of MAPK14 (**b**). ^**^P < 0.01, ^***^P < 0.001, ^****^P < 0.0001 compared by one-way ANOVA (*n* = 3). dauriporphine: 10 µM dauriporphine; miR-inhibitor NC: 20 nmol miR-424-5p inhibitor negative control; miR-inhibitor: 50 nmol miR-424-5p inhibitor; si-MAPK14 NC: 50 nmol small interference RNA of MAPK14 negative control; si-MAPK14: 50 nmol small interference RNA of MAPK14

MAPK14 was predicted to show several binding sites with miR-424-5p, and its luciferase reporter activity was negatively regulated by miR-424-5p (Fig. 3a). Dauriporphine induced significant downregulation of MAPK14 in A549 cells (Fig. 3b). Silencing miR-424-5p improved the expression of MAPK14 in dauriporphine-treated A549 cells, which was reversed by the transfection of MAPK14 siRNA (Fig. 3b).

**Fig. 4:**
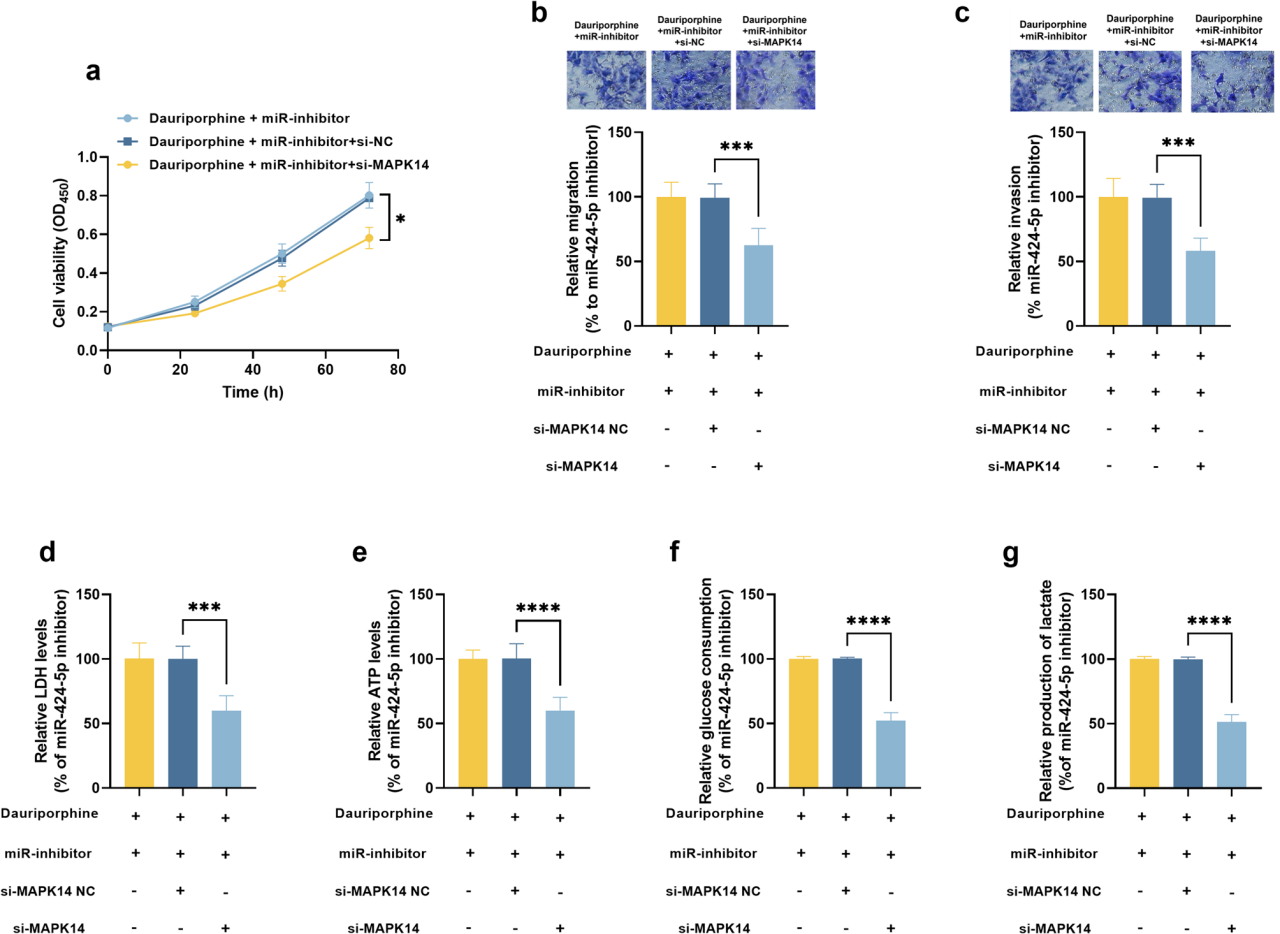
Involvement of MAPK14 in the protective effect of miR-424-5p on dauriporphine-treated A549 cells. a-e. cell viability (**a**), migration (**b**), invasion (**c**), ATP level (**d**), LDH level (**e**), glucose consumption (**f**), and lactate production (**g**) regulated by the miR-424-5p/MAPK14 axis in dauriporphine-treated A549 cells. ^*^P < 0.05, ^***^P < 0.001, ^****^P < 0.0001 compared by one-way ANOVA (*n* = 3). dauriporphine: 10 µM dauriporphine; miR-inhibitor: 50 nmol miR-424-5p inhibitor; si-MAPK14 NC: 50 nmol small interference RNA of MAPK14 negative control; si-MAPK14: 50 nmol small interference RNA of MAPK14

In the presence of miR-424-5p inhibition, silencing MAPK14 significantly reduced the viability (Fig. 4a), migration (Fig. 4b), and invasion (Fig. 4c) of A549 cells. The ATP (Fig. 4d), LDH (Fig. 4e), glucose consumption (Fig. 4f), and lactate production (Fig. 4g) levels were also improved by the knockdown of MAPK14 in dauriporphine-treated A549 cells, indicating the recovered energy metabolism.

## Discussion

This study demonstrated the inhibitory effect of dauriporphine on the viability, motility, and energy metabolism of lung cancer cells, and the miR-424-5p/MAPK14 axis was revealed as the downstream molecular mechanism.

Dauriporphine is a kind of isoaporphine alkaloid primarily extracted from the rhizome of *Manispernum daericum* DC. With the deepening understanding of *Manispernum daericum* DC. extraction, dauriporphine has been revealed to show various pharmacological activities, where its anti-tumor activity attracts special attention [34, 35]. Although the potential of dauriporphine in treating lung cancer has not been reported, there have been several alkaloids revealed to suppress lung cancer through distinct molecular mechanisms [34, 36, 37]. Herein, the anti-lung cancer effect of dauriporphine has been revealed with the employment of a lung adenocarcinoma cell. Inhibition of cell growth and motility was observed under the treatment of dauriporphine, and the inhibitory effect was demonstrated to be concentration-dependent. Cell growth and motility are closely related to the tumor progression and metastasis, of which the inhibition could effectively suppress the development of lung adenocarcinoma [38, 39]. The glycolysis pathway has been evidenced as the main means to generate energy for tumor cell growth [40]. During the glycolysis process, a series of enzymatic reactions produced LDH and ATP under anaerobic conditions. ATP levels are the direct substances supplying energy and could indicate the cellular capacity of energy reserve and immediate energy supply [41]. The dynamic changes in APT levels could indicate the metabolic activity and proliferation capacity of tumor cells [42]. While LDH is a terminal enzyme of the glycolytic pathway. The production of lactate and the consumption of glucose are also associated with the energy metabolism of tumor cells [43]. Under some tumor conditions, the lactate levels were associated with the survival rate and recurrence risk of cancer patients [44–46]. The accumulation of LDH and lactate would induce the decreasing pH, which exerted protective effects on tumor cells, resulting in immune escape [47]. It was reported that the acid environment could promote the invasion and immune escape of tumor cells and improve their resistance to radiotherapy and chemotherapy [48, 49]. The ATP and LDH levels were significantly suppressed by dauriporphine, indicating the adverse microenvironment for tumor cells exerted by dauriporphine. Hence, dauriporphine can be considered a potential anti-lung cancer compound for drug development and clinical therapies. However, the metabolic diversity of tumor cells was associated with various environmental factors, such as mitochondrial function and expression of related genes [50–52]. Therefore, the response of ATP and LDH levels to dauriporphine should be further validated with expanding metabolic parameters, which could help understand the underlying regulatory mechanisms.

Previous studies have identified a number of miRNAs as potential biomarkers for the progression and prognosis of lung adenocarcinoma [53–56]. miR-424-5p was identified as a tumor regulator in various human cancers, such as bladder cancer and oral squamous cell carcinoma [57, 58]. In lung cancer, miR-424-5p was considered of great potential in tumor progression and has been illustrated to mediate the regulation of non-small cell lung cancer development by lncRNAs, implying its potential to serve as a therapeutic target [29, 59, 60]. Significant downregulation of miR-424-5p was observed in H1299 and A549 cells relative to normal lung epithelium cells, which is consistent with previous reports [61]. Overexpression of miR-424-5p showed significant inhibitory effects on cell viability and motility of A549 cells, indicating its tumor suppressor role. Dauriporphine induced significantly increasing miR-424-5p levels in A549 cells while silencing miR-424-5p could reverse the anti-tumor effect of dauriporphine on A549 cells. Hence, miR-424-5p was considered to mediate the inhibitory effect of dauriporphine on A549 cells.

Based on previous pharmacological network results, the potential involvement of MAPK14 in the anti-tumor effect of dauriporphine and the regulatory effect of miR-424-5p was raised. MAPK14 was previously reported to predict the risk, chemotherapy toxicity, and tumor progression in non-small cell lung cancer [30, 31, 62] and was identified as a critical target of various anti-tumor herbal compounds, such as Andrographis and Polygonatum cyrtonema Hua [63, 64]. The targeting relationship between miR-424-5p and MAPK14 was revealed in LPS-induced lung injury, which was also confirmed in lung adenocarcinoma cells in the present study [33]. Silencing miR-424-5p significantly elevated the expression of MAPK14 in dauriporphine-treated A549 cells, while downregulating MAPK14 not only reversed the effect of miR-424-5p on its expression but also reversed the protective effect of miR-424-5p on dauriporphine-treated lung adenocarcinoma cells. Therefore, dauriporphine was hypothesized to inhibit the progression of lung adenocarcinoma through the miR-424-5p/MAPK14 axis.

This study is a primary mechanism investigation, revealing the underlying mechanism of dauriporphine inhibiting lung adenocarcinoma progression on the cell level. Due to the limited finical, the effect of dauriporphine and its potential mechanism were only confirmed in one cell line, which needs further validation in other tumor cells. Additionally, the effect of dauriporphine on non-cancerous cells would provide a theoretical reference for the therapeutic safety of dauriporphine, which would also attract special attention in future investigations. There is a need for clinical trials to estimate the specific significance of dauriporphine in the clinical management of lung adenocarcinoma.

## Conclusion

Dauriporphine suppressed lung adenocarcinoma cell function. The anti-lung cancer effect of dauriporphine was displayed through upregulation of miR-424-5p, which inhibits the effect of MAPK14 in cancer progression. Dauriporphine can be considered in drug development for lung adenocarcinoma, with the miR-424-5p/MAPK14 axis as the therapeutic target. Additionally, more components of various herbs targeting miR-424-5p can be explored to provide more therapeutic strategies for human cancers.

## Electronic supplementary material

Below is the link to the electronic supplementary material.

Supplementary Material 1

Supplementary Material 2

## Data Availability

The datasets used and/or analysed during the current study are available from the corresponding author on reasonable request.
