# Supplementary material for: Dauriporphine inhibited lung cancer cell viability, motility, and energy metabolism through the miR-424-5p/MAPK14 axis
Source: Hereditas. 2025 Jun 11;162:101. doi: 10.1186/s41065-025-00473-w (PMC12153179; doi:10.1186/s41065-025-00473-w)
Supplement: Supplementary file 1 — Supplementary Material 1 [file 41065_2025_473_MOESM1_ESM.docx]

| primer | Sequences |
| --- | --- |
| miR-424-5p forward | 5'-AGCAGCAATTCATGTTTTG-3' |
| miR-424-5p reverse | 5'-GAACATGTCTGCGTATCTC-3' |
| U6 forward | 5'-GCTTCGGCAGCACATATACTAAAAT-3' |
| U6 reverse | 5'-CGCTTCACGAATTTGCGTGTCAT-3' |
| MAPK14 forward | 5'-CGAGCGATACCAGAACCTGT-3' |
| MAPK14 reverse | 5'-GCGTGAATGATGGACTGAAA-3' |
| GAPDH forward | 5'-ATTCCATGGCA CCGTCAAGGCTGA-3' |
| GAPDH reverse | 5'-TTCTCCATGGTG GTGAAGACGCCA-3' |

Table S1. Primer sequences used in PCR assay
