# Supplementary figures and images for: Dauriporphine inhibited lung cancer cell viability, motility, and energy metabolism through the miR-424-5p/MAPK14 axis
Source: Hereditas. 2025 Jun 11;162:101. doi: 10.1186/s41065-025-00473-w (PMC12153179; doi:10.1186/s41065-025-00473-w)

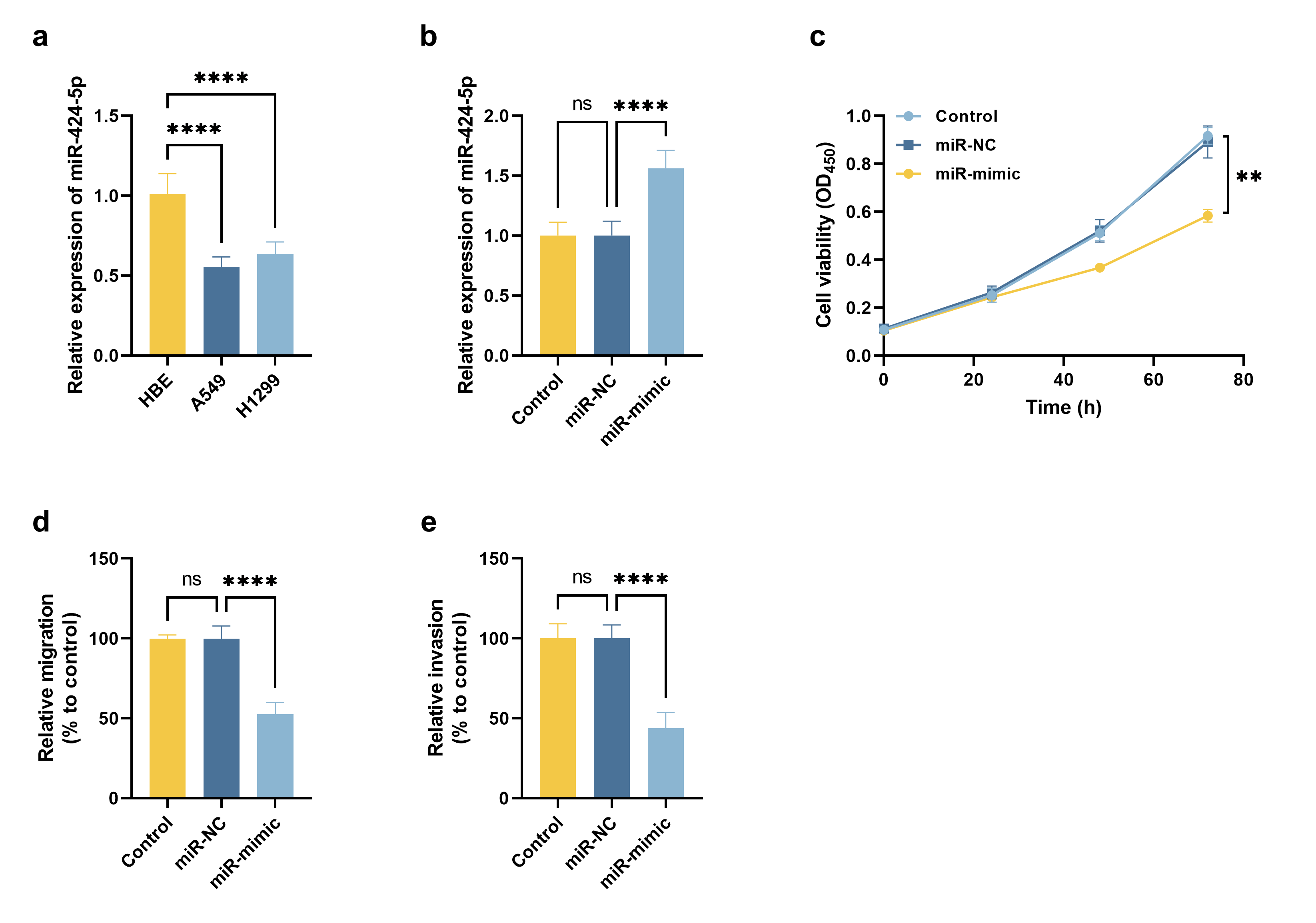

Supplement: Supplementary file 2 — Supplementary Material 2 [file 41065_2025_473_MOESM2_ESM.tif]
